# Biochemical Basis of Cyfluthrin and DDT Resistance in *Anopheles stephensi* (Diptera: Culicidae) in Malarious Area of Iran

**Published:** 2018-09-30

**Authors:** Mohammad Amin Gorouhi, Mohammad Ali Oshaghi, Hassan Vatandoost, Ahmad Ali Enayati, Ahmad Raeisi, Mohamad Reza Abai, Yaser Salim-Abadie, Ahmad Ali Hanafi-Bojd, Azim Paksa, Fatemeh Nikpoor

**Affiliations:** 1 Department of Vector Biology and Control, School of Health, Kerman University of Medical Sciences, Kerman, Iran; 2 Department of Medical Entomology and Vector Control, School of Public Health, Tehran University of Medical Sciences, Tehran, Iran; 3 Dept. of Chemical Pollutants and Pesticides, Institute for Environmental Research, Tehran University of Medical Sciences, Tehran, Iran; 4 Mazendaran University of Medical Sciences, Sari, Mazendaran, Iran; 5 National Programme Manager for Malaria Control, Ministry of Health, Tehran, Iran; 6 Department of Health Service and Health Promotion, School of Health, Rafsanjan University of Medical Sciences, Rafsanjan, Iran

**Keywords:** *Anopheles stephensi*, Insecticide, Resistance mechanisms, Malaria

## Abstract

**Background:**

*Anopheles stephensi* is a key urban malaria vector in the Indian subcontinent and Middle East including south and southeast of Iran. Wide application of insecticides resulted in resistance of this species to various insecticides in these regions. This study was conducted to reveal the role of metabolic mechanisms in the development of resistance in *An. stephensi* to DDT and cyfluthrin.

**Methods:**

Field mosquito specimens were collected from Chabahar Seaport, southeast corner of Iran, in 2015. Insecticide susceptibility and enzyme assays were conducted as recommended by WHO.

**Results:**

Mean enzyme ratios were 3.95 and 3.04 for α- esterases and 2.40 and 1.97 for β- esterases in the DDT and cyfluthrin- resistant populations correspondingly compared with the susceptible strain. The GSTs enzyme mean activity ratios were 5.07 and 2.55 in the DDT and cyfluthrin- resistant populations compared with the susceptible beech strain. The cytochrome p450s enzyme ratios were 1.11 and 1.28 in the DDT and cyfluthrin- resistant populations respectively compared with the susceptible beech strain.

**Conclusion:**

Metabolic mechanisms play a crucial role in the development of DDT and cyfluthrin resistance in *An. stephensi*, therefore, further evaluation of the mechanisms involved as well as implementation of proper insecticide resistance management strategies are recommended.

## Introduction

Malaria is still a major public health problem in southeast corner of Iran (1). There are seven *Anopheles* species as malaria vectors in Iran including *An. stephensi*, *An. culcifacies* s.l., *An. maculipennis* s.l., *An. sacharovi*, *An. superpectus* s.l., *An. dthali*, and *An. fluviatilis* s.l.. *Anopheles stephensi* is the most important malaria vector in southern region of the country (2–10).

Application of chemical insecticides is one of the most important interventions for malaria control, used in Iran during past decades. Different groups of insecticides including organochlorines (DDT, dieldrin and BHC), organophosphates (pirimiphos-methyl and malathion), carbamate (propoxur) and pyrethroids (lambdacyhalothrin and delthamethrin) in different forms of application such as indoor residual spraying (IRS) and insecticide-treated nets (ITNS) for adult mosquito control and organophosphates for larviciding were used in malarious areas of the country (11–13).

Iran has embarked on the malaria elimination program since 2007 relying on application of chemical insecticides specially pyrethroid compounds for malaria vector control (14).

*Anopheles stephensi* is resistant to several insecticides including DDT, dieldrin, and malathion (12, 15–19). The first indication of pyrethroid resistance was reported from Chabahar Seaport, southeast of Iran in 2012 (13). Moreover, there are many reports on resistance of this species to different insecticide groups including pyrethroids from Iran neighboring countries including Pakistan, Afghanistan, the Indian subcontinent as well as the Middle East countries (14, 15, 18, 20–25).

Due to the importance of pyrethroids in malaria control program and the slow process of development of new insecticide compounds, monitoring and management of insecticide resistance are necessary (26, 27). Metabolic and target site insensitivity are two common resistant mechanisms in insects. In metabolic resistance, alteration in the levels or activities of detoxification enzymes such as esterases, glutathione S-transferases (GSTs), and cytochrome P450s may occur (24, 28). In target site insensitivity, mutations in the sodium channel, acetylcholinesterase and GABA receptor genes occur (29). Therefore, determination of resistance mechanisms in *An. stephensi* is essential for proper management of insecticide resistance through vector control interventions.

The aim of this study was to determine the possible involvement of enzymes groups’ in DDT and pyrethroid insecticides resistance functioning in *An. stephensi*, the main malaria vector in southeast of Iran.

## Materials and Methods

### Mosquito collection and rearing

**Fig. 1.**
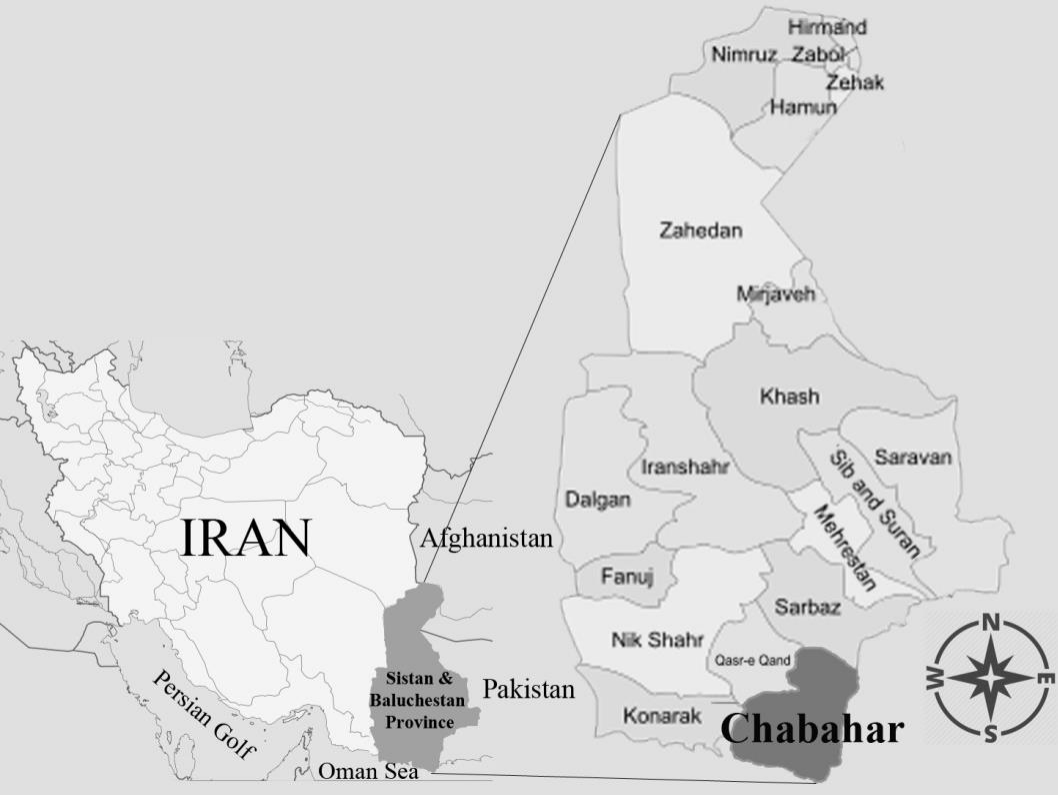
The map of the study area in Chabahar Seaport, Southeast of Iran

*Anopheles stephensi* larvae were collected from larval habitats using the standard dipper from Chabahar Seaport (25°25′N, 60°45′E) Sistan and Baluchestan Province, southeast of Iran (Fig. 1) during Apr to June 2015. The larvae specimens were transported in cool boxes to insectary of the Medical Entomology and Vector Control Department, School of Public Health, Tehran University of Medical Sciences, Tehran, Iran and reared to adult stage under standard condition at 25 °C, 80% relative humidity with a 12h day/night lighting cycle. The adult mosquito specimens were identified to species level using the identification key (30).

Moreover, a pyrethroid susceptible strain (Beech strain) originated in India in 1940 and kept in the insectary without being exposed to insecticides used as a control in all experiments.

### Adult susceptibility tests and selection

Six different insecticide impregnated papers including DDT 4%, lambdacyhalothrin 0.05%, deltamethrin 0.05%, cyfluthrin 0.15%, permethrin 0.75%, and etofenprox 0.5% supplied by WHO were used for evaluating the susceptibility status of *An. stephensi* populations from Chabahar. Two or three days old adult female mosquitos that were kept on 10% aqueous sucrose solution were used for susceptibility test procedure according to the WHO method (27). Then the mosquito populations with the lowest mortality rates were subjected to selection pressure of the two insecticides in the laboratory. The mosquito populations were exposed to the two insecticides in two separate lines over 18 and 19 generations throughout four and five selection phases. For both insecticides, mortality rate was calculated in different times and regression lines were plotted in each generation using Microsoft Excel (ver. 2013). A subset (40 specimens for each sample) of resistant and susceptible mosquito populations were placed in a 1.7ml tubes and kept in freezer (−80 °C). These frozen specimens were then transported in a cold chain to the Pesticide Biochemistry Laboratory of Medical Entomology Department, School of Public Health, Mazandaran University of Medical Sciences, Sari, Iran for further biochemical assays.

### Biochemical assays

Biochemical tests were performed according to the method described by Hemingway (1998) (31). The enzyme activities/contents of P450s, glutathione S-transferases (GSTs), and esterases using corresponding fresh buffer solutions were quantified. Frozen adult mosquito specimens were individually put in wells of flat-bottomed 96-well microtiter plate and manually homogenized using a steel pestle in 250μL cold distilled water at 4 °C. The plate was spun at 3000rpm for 20min in a Beckman Coulter (Beckman Inc., USA) centrifuge at 4 °C and the supernatant was used as the source of enzymes in reaction mixtures. In each biochemical assay, blank replications (all component of the reaction mixture except for the enzyme source) were provided. Preparation of all reaction mixtures was carried out on ice (31).

### Total Protein assay

In order to minimize the error due to different size and protein contents of mosquitos and homogenizing process, total protein component of each specimen was measured using Bradford method in triplicate by adding 300 μL of Bio-Rad solution (diluted with distilled water by 1:4) to 10μL of the homogenate. After 5min incubation at room temperature, the absorbance was measured at 570nm in a Biotek ELX808 Ultra Microplate Reader (Biotek Inc, USA) (31). The value was changed into product concentration using a bovine serum albumin standard curve obtained with the same reagents and method.

### Cytochrome P450s assay

This test quantifies the amount of hem containing protein in the specimens. In each well, the reaction cocktail comprised of 20μl of the mosquito homogenate in duplicate, 80μL of 0.0625M potassium phosphate buffer PH 7.2, 200μL of 3, 3’, 5, 5’ tetramethyl benzidine (TMB) solution (0.01g TMB dissolved in 5ml methanol plus 15ml of 0.25M sodium acetate buffer pH 5.0) and 25μL of 3% hydrogen peroxide. After 2h incubation in room temperature, the absorbance was measured at 450nm. The protein contents were described as correspondent units of cytochrome (EUC) P450sec/mg protein corrected for the known hem content of P450s and cytochrome C using a standard curve of purified cytochrome C (31).

### Glutathione S-transferase assay

The reaction mixture contained 200μL of reduced glutathione plus 1-coloro-2, 4-dinitrobenzene (CDNB) added to 10μL of the mosquito homogenate in duplicate. The increase in absorbance was measured at 340nm for 5 min. The amount of conjugate produced/min/mg protein (mM) using the extinction coefficient of CDNB corrected for the path length of the solution in the microplate well was reported as enzyme activity (31).

### General esterase assay

In this assay, the activity of α-esterase and β-esterase with the alfa and beta-naphthyl acetate as universal substrates were measured. To a reaction mixture of 200μL of alpha or beta-naphthyl acetate solution was added to 20μL of mosquito homogenate in duplicate. After 30min incubation at room temperature, 50μL of fast blue solution was added to each mixture. Plates were incubated at room temperature for another 5min and then absorbance was recorded at 570nm (19). The optical densities (OD) of solutions were converted to product concentration as μM of product formed/min/mg protein using standard curves of ODs for known concentrations of the products α- or β –naphthol (31).

In each biochemical assays, four blank replicates were set using the same materials of each assay except for distilled water added instead of the mosquito homogenate. The ODs of the wells containing mosquito homogenates were adjusted by deducting with the average ODs of the blank replicates.

### Data conversion and analyses

The activity/contents of the enzymes were measured and used for further analysis by Microsoft Excel. The data then were transformed into the actual enzyme activity values using standard curves. Mean values of the enzyme activities of all populations were compared using ANOVA in conjunction with the Tukey’s statistical test using SPSS ver. 19 software (Chicago, IL, USA) (P< 0.05). Enzyme ratios (ER) were computed by dividing the mean activities of each resistant population with those of the Beech susceptible strain (24).

## Results

### Selection process

**Fig. 2.**
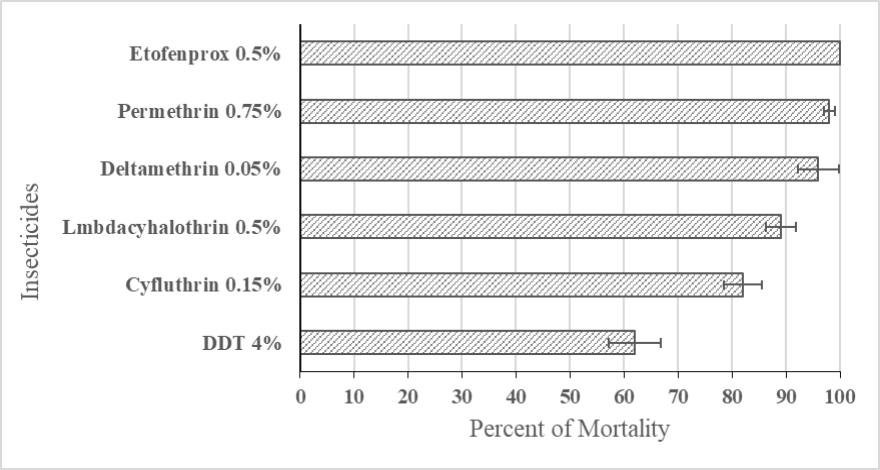
Mortality rate (mean and SEM) of *Anopheles stephensi* Chabahar strain to six insecticides

Susceptibility tests showed that *An. stephensi* Chabahar strain was susceptible to permethrin and etofenprox, resistant candidate to deltamethrin and resistant to DDT, cyfluthrin, and lambda cyhalothrin. This strain showed the highest resistance to DDT 4% and cyfluthrin 0.15% respectively among the insecticides tested (Fig. 2). Populations with resistance ratio (RR) to Cyfluthrin of 11.6 and to DDT of 2.05 RR in comparison with the susceptible strain were chosen for insecticide selection process. This process continued for four and five phases throughout 18 and 19 generations respectively to achieve resistance ratio of 28.75 for the population exposed to DDT (R1) and 6.8 for the population exposed to cyfluthrin (R2).

### Biochemical assays

**Fig. 3.**
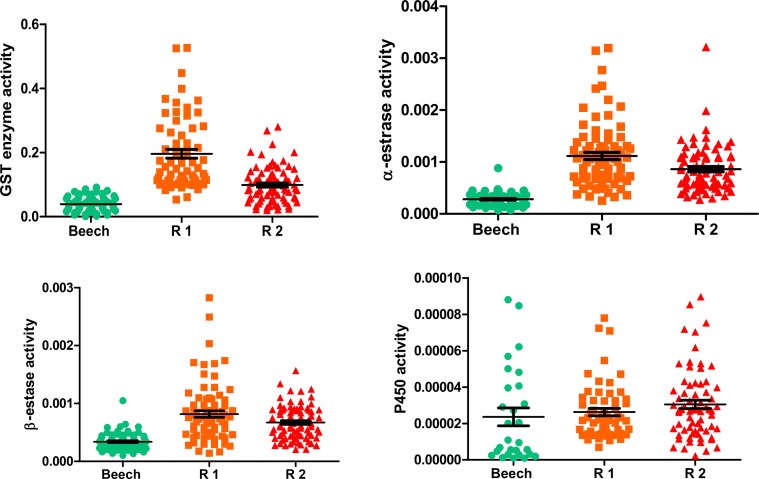
Mean and SEM activity profiles of P450, GST and α- and β-Esterase enzymes in the DDT resistant (R1), the Cyfluthrin resistant (R2), and the susceptible Beech-strain of *Anopheles stephensi*

Activities of α- and β-esterases, glutathione-S-transferase (GST) and the contents of cytochrome P450s were tested for the R1 and R2 *An. stphensi* populations are summarized in Table 1 and Fig. 3. The cytochrome P450s enzyme ratios were 1.11 and 1.28 fold in the DDT and cyfluthrin- resistant populations compared with the susceptible strain. Although the median activities of the resistant population were 2.2 and 2.7 times more than the susceptible one, however, the mean activity/content of P450 enzymes in the resistant and the susceptible strains was not significant (P< 0.05). The enzyme ratios for esterases with α-naphthyl acetate were 3.95 and 3.04 and with β-naphthyl acetate were 2.4 and 1.97 in the DDT and cyfluthrin- resistant populations correspondingly compared with the susceptible strain. The GSTs enzyme ratios were 5.07 and 2.55 in the DDT and cyfluthrin- resistant populations compared with the susceptible Beech strain. The enzyme ratios for both the esterases and GST enzymes were higher in the DDT-resistant population than the cyfluthrin- resistant population (Fig. 3). Statistical analysis showed that the activity/content of the two esterases and GST enzymes of the selected populations and the beech susceptible strain were significantly different (P< 0.05) (Fig. 3).

**Table 1.** Details of enzyme activities and enzyme ratios (ER) measured in *Anopheles stephensi* resistant populations from southeastern Iran. Beech, susceptible, R1, DDT resistant, and R2, the Cyfluthrin resistant population

**Enzyme**	**Population**	**N**	**Median**	**Mean±SE**	**Enzyme Ratio**
**P450**	**Beech**	53	1.014e-005	2.365e-005±4.899e-006	1
	**R1**	58	2.183e-005	2.626e-005±2.035e-006	1.11
	**R2**	69	2.753e-005	3.050e-005±2.319e-006	1.28
**GST**	**Beech**	71	0.03485	0.03856±0.002610	1
	**R1**	66	0.15330	0.19590±0.01373	5.07
	**R2**	69	0.08683	0.09857±0.006261	2.55
**α-eseterase**	**Beech**	80	0.0002753	0.0002825±1.307e-005	1
	**R1**	80	0.001022	0.001116±6.949e-005	3.95
	**R2**	80	0.0007664	0.0008604±5.001e-005	3.04
**β-eseterase**	**Beech**	80	0.0003146	0.0003397±1.588e-005	1
	**R1**	80	0.0007474	0.0008167±5.603e-005	2.4
	**R2**	79	0.0006188	0.0006718±3.299e-005	1.97

## Discussion

This study revealed that *An. stephensi* from Chabahar District, southeast of Iran is resistant to pyrethroids including cyfluthrin and lambda cyhalothrin, DDT and tolerant to deltamethrin. Therefore, far various modes of resistance including modification or overexpression of detoxification enzymes, target site insensitivity, as well as behavioral adaptations have been developed and documented in insects (32, 33). Resistance to insecticides might be due to increased monitoring, misuse of insecticides, geographical extension of resistance, and new resistance genes (34) leading to decrease in the effectiveness of vector control programs. Resistance to pyrethroids in *An. stephensi* has been reported in several countries in the Eastern Mediterranean Region, notably Afghanistan, and Oman (12, 15, 16, 24, 34, 35). In addition, there are reports on DDT resistance in Yemen (34, 35), DDT and pyrethroid resistant in *Anopheles* mosquito of Iran (13, 15).

There have been reports of resistance to three of the four insecticides classes in *An. stephensi* mosquitoes in Afghanistan (24, 34). Resistances to all four classes of insecticide have been reported in *An. stephensi* from Somalia and Sudan, including widespread resistance to DDT and an increasing frequency of resistance to pyrethroids (34, 36). Unfortunately, most of the new resistance reports are to pyrethroid compounds that are the only insecticides used for long lasting insecticide nets (LLINs).

This study showed that biochemical mechanisms are driving the resistance in this field population. This was shown by measuring the activities of the enzymes which could be responsible for the insecticide resistance in *An. stephensi* from Chabahar. The differences between activities of three enzyme groups including alpha and beta esterases, and GSTs in the Chabahar population were higher than those of the susceptible Beech strain, esterases and GSTs could all be involved in insecticide resistance in this population. Our result showed that in order α-esterase, β-esterase and GST enzyme have played the highest role in resistance to the DDT resistant populations (Table 1). This order was α-esterase, GST enzyme, and then β-esterase for the cyfluthrin resistant population. Accordingly, almost similar situation has been reported in the field populations of *An. stephensi* in Afghanistan, a neighboring country sharing border line with Chabahar (24). Esterases and cytochrome P450s are involved in pyrethroid resistance in *An. stephensi* (21, 37), *An. gambiae* (38), *An. albimanus* (39, 40), and *An. minimus* (41). Moreover, esterases are involved in organophosphate (OP) resistance with cross-resistance to pyrethroids (21, 40, 42, 43). Rising enzyme activities in many insects have been reported including mosquitoes which are resistant to various insecticides from different parts of the world (17, 28, 42, 44–47). As biochemical mechanisms are involved in insecticide resistance in the Chabahar population, using of synergists in formulation of pyrethroid insecticides should be evaluated.

Different forms of resistance mechanisms have been reported in different species of *Anopheles* so that in some species only metabolic resistance has currently been reported. For example in the study on *An. funestus s.s* in Uganda just enzymatic resistant have been reported (48), whereas both metabolic and target-site insensitivity have been found as resistant mechanisms in *An. gambiae s.s.* in Africa (48, 49).

In this study the target-site insensitivity (kdr) mutations as a potential resistance mechanism in the *An. stephensi* populations were not examined. These mutations have been shown in *An. stephensi* from Afghanistan (24) and might be present in Chabahar population. Therefore this molecular assay is highly recommended to test the presence of kdr resistance mechanism in this population.

## Conclusion

The *An. stephensi* Chahbahar population is becoming resistant to deltamethrin. This insecticide is currently used in malaria elimination program against malaria vectors including *An. stephensi* in the region. Although this insecticide may still be useful to combat *An. stephensi* in the area, surveillance of the susceptibility of populations by bioassay as well as biochemical and molecular assays are recommended to prevent building up of deltamethrin resistance levels. Insecticide resistance management strategies are also recommended to suspend or to slow the rate of resistance development to deltamethrin in Chahbahar District.

This study showed enzyme elevation and enzymatic resistance in the resistant population. Therefore conducting biochemical assays along with bioassay can be helpful for monitoring and management of resistant phenomena. Biochemical assays can be involved in routine malaria program for better monitoring and management of resistance in vector populations. Moreover, using other insecticides with different mode of action can be helpful for vector resistant management.

The main resistance mechanism in *An. stephensi* from the study area is metabolic and different enzyme groups play various roles in the resistance. Therefore, continuous surveillance of the susceptibility of populations and monitoring of insecticide resistance in the malaria vectors is crucial for successful control measures in Iran.

## References

[1] Norouzinejad F Ghaffari F Raeisi A (2016) Epidemiological status of malaria in Iran, 2011–2014. Asian Pac J Trop Med. 9: 1055–1061. 27890364 10.1016/j.apjtm.2016.09.007

[2] Azari-Hamidian S (2011) Larval habitat characteristics of the genus Anopheles (Diptera: Culicidae) and a checklist of mosquitoes in Guilan Province, northern Iran. Iran J Arthropod Borne Dis. 5(1): 37–53. 22808409 PMC3385571

[3] Mehravaran A Oshaghi M Vatandoost H Abai M Ebrahimzadeh A Roodi AM Grouhi A (2011) First report on *Anopheles fluviatilis* U in southeastern Iran. Acta Trop. 117: 76–81. 20933492 10.1016/j.actatropica.2010.09.011

[4] Naddaf Dezfouli S Oshaghi M Vatandoost H Assmar M (2003) rDNA-ITS2 based species-diagnostic polymerase chain reaction assay for identification of sibling species of *anopheles fluviatilis* in Iran. Southeast Asian J Trop Med Public Health. 34: 56–60. 19230572

[5] Vatandoost H Oshaghi M Abaie M Shahi M Yaaghoobi F Baghaii M Hanafi-Bojd AA Zamani G Townson H (2006) Bionomics of *Anopheles stephensi* Liston in the malarious area of Hormozgan Province, southern Iran, 2002. Acta Trop. 97: 196–203. 16329986 10.1016/j.actatropica.2005.11.002

[6] Karimian F Oshaghi MA Sedaghat MM Waterhouse RM Vatandoost H Hanafi-Bojd AA Ravasan NM Chavshin AR (2014) Phylogenetic analysis of the oriental-palearctic-afrotropical members of anopheles (Culicidae: Diptera) based on nuclear rDNA and mitochondrial DNA characteristics. Jpn J Infect Dis. 67: 361–367. 25241686 10.7883/yoken.67.361

[7] Oshaghi M Shemshad K Yaghobi-Ershadi M Pedram M Vatandoost H Abai M Abai MR Akbarzadeh K Mohtarami F (2007) Genetic structure of the malaria vector *Anopheles superpictus* in Iran using mitochondrial cytochrome oxidase (COI and COII) and morphologic markers: A new species complex? Acta Trop. 101: 241–248. 17367742 10.1016/j.actatropica.2007.02.006

[8] Oshaghi M Vatandoost H Gorouhi A Abai M Madjidpour A Arshi S Sadeghi H Nazari M Mehravaran A (2011) Anopheline species composition in borderline of Iran-Azerbaijan. Acta Trop. 119: 44–449. 21513694 10.1016/j.actatropica.2011.04.005

[9] Oshaghi M Yaghobi-Ershadi M Shemshad K Pedram M Amani H (2008) The *Anopheles superpictus* complex: introduction of a new malaria vector complex in Iran. Bull Soc Pathol Exot. 101: 429–434. 19192616

[10] Sedaghat M Linton YM Oshaghi M Vatandoost H Harbach R (2003) The *Anopheles maculipennis* complex (Diptera: Culicidae) in Iran: molecular characterization and recognition of a new species. Bull Entomol Res. 93: 527–535. 14704099 10.1079/ber2003272

[11] Feo ML Eljarrat E Manaca MN Dobaño C Barcelo D Sunyer J Alonso PL Menendez C Grimalt JO (2012) Pyre-throid use-malaria control and individual applications by households for other pests and home garden use. Environ Int. 38: 67–72. 21982035 10.1016/j.envint.2011.08.008

[12] Gorouhi MA Vatandoost H Oshaghi MA Raeisi A Enayati AA Mirhendi H Hanafi-Bojd AA Abai MR Salim-Abadi Y Rafi F (2016) Current susceptibility status of *Anopheles stephensi* (Diptera: Culicidae) to different imagicides in a malarious area, southeastern of Iran. J Arthropod Borne Dis. 10(4): 493–500. 28032101 PMC5186739

[13] Vatandoost H Hanafi-Bojd AA (2012) Indication of pyrethroid resistance in the main malaria vector, *Anopheles stephensi* from Iran. Asian Pac J Trop Med. 5: 722–726. 22805725 10.1016/S1995-7645(12)60114-X

[14] Raeisi A Gouya MM Nadim A Ranjbar M Hasanzehi A Fallahnezhad M Sakeni M Safari R Saffari M Mashyekhi M Ahmadi Kahnali A Mirkhani V Almasian E Faraji L Paktinat Jalali B Nikpour F (2013) Determination of malaria epidemiological status in Iran's malarious areas as baseline information for implementation of malaria elimination program in Iran. Iran J Public Health. 42: 326–333. 23641411 PMC3633804

[15] Abai M Mehravaran A Vatandoost H Oshaghi MA Javadian E Mashayekhi M Mosleminia A Piyazak N Edallat H Mohtarami F Jabbari H Rafi F (2008) Comparative performance of imagicides on *Anopheles stephensi*, main malaria vector in a malarious area, southern Iran. J Vector Borne Dis. 45: 307–312. 19248658

[16] Nejati J Vatandoost H Oshghi MA Salehi M Mozafari E Moosa-Kazemi SH (2013) Some ecological attributes of malarial vector *Anopheles superpictus* Grassi in endemic foci in southeastern Iran. Asian Pac J Trop Biomed. 3: 1003–1008. 24093794 10.1016/S2221-1691(13)60193-8 PMC3805098

[17] Soltani A Vatandoost H Oshaghi MA Ravasan NM Enayati AA Asgarian F (2015) Resistance Mechanisms of *Anopheles stephensi* (Diptera: Culicidae) to Temephos. J Arthropod Borne Dis. 9: 71–83. 26114145 PMC4478420

[18] Syafruddin D Hidayati AP Asih PB Hawley WA Sukowati S Lobo NF (2010) Detection of 1014F kdr mutation in four major Anopheline malaria vectors in Indonesia. Malar J. 9: 315–323. 21054903 10.1186/1475-2875-9-315 PMC2989330

[19] Soltani A Vatandoost H Oshaghi MA Enayati AA Raeisi A Eshraghian MR Soltan-Dallal MM Hanafi-Bojd AA Abai MR Rafi F (2013) Baseline susceptibility of different geographical strains of *Anopheles stephensi* (Diptera: Culicidae) to temephos in malarious areas of Iran. J Arthropod Borne Dis. 7: 56–56. 23785695 PMC3684497

[20] Chen L Zhong D Zhang D Shi L Zhou G Gong M Zhou H Sun Y Ma L He J Hong S Zhou D Xiong C Chen C Zou P Zhu C Yan G (2010) Molecular ecology of pyrethroid knockdown resistance in *Culex pipiens* pallens mosquitoes. PLoS One. 5(7): e11681. 20657783 10.1371/journal.pone.0011681 PMC2908134

[21] Enayati AA Vatandoost H Ladonni H Townson H Hemingway J (2003) Molecular evidence for a kdr-like pyrethroid resistance mechanism in the malaria vector *mosquito Anopheles stephensi*. Med Vet Entomol. 17: 138–44. 12823830 10.1046/j.1365-2915.2003.00418.x

[22] Hemingway J (1983) The genetics of malathion resistance in *Anopheles stephensi* from Pakistan. Trans R Soc Trop Med Hyg. 77: 106–8. 6679355 10.1016/0035-9203(83)90030-5

[23] Lol JC Castellanos ME Liebman KA Lenhart A Pennington PM Padilla NR (2013) Molecular evidence for historical presence of knock-down resistance in *Anopheles albimanus*, a key malaria vector in Latin America. Parasit Vectors. 6: 268–278. 24330978 10.1186/1756-3305-6-268 PMC3848997

[24] Safi NHZ Ahmadi AA Nahzat S Ziapour SP Nikookar SH Fazeli-Dinan M Enayati A Hemingway J (2017) Evidence of metabolic mechanisms playing a role in multiple insecticides resistance in *Anopheles stephensi* populations from Afghanistan. Malar J. 16: 100–107. 28253925 10.1186/s12936-017-1744-9 PMC5335852

[25] Singh OP Dykes CL Lather M Agrawal OP Adak T (2011) Knockdown resistance (kdr)-like mutations in the voltage-gated sodium channel of a malaria vector *Anopheles stephensi* and PCR assays for their detection. Malar J. 10: 59. 21401946 10.1186/1475-2875-10-59 PMC3068127

[26] Coleman M Hemingway J Gleave KA Wiebe A Gething PW Moyes CL (2017) Developing global maps of insecticide resistance risk to improve vector control. Malar J. 16: 86. 28222727 10.1186/s12936-017-1733-z PMC5320685

[27] World Health organization (2013) Test procedures for insecticide resistance monitoring in malaria vector mosquitoes. Geneva, World Health Organization.

[28] Enayati AA Ranson H Hemingway J (2005) Insect glutathione S-transferases and insecticide resistance Insect Mol Biol. 14: 3–3. 15663770 10.1111/j.1365-2583.2004.00529.x

[29] Hemingway J Hawkes NJ McCarroll L Ranson H (2004) The molecular basis of insecticide resistance in mosquitoes. Insect Biochem Mol Biol. 34: 653–665. 15242706 10.1016/j.ibmb.2004.03.018

[30] Azari-Hamidian S Harbach RE (2009) Keys to the adult females and fourth-instar larvae of the mosquitoes of Iran (Diptera: Culicidae). Zootaxa. 2078: 1–33.

[31] Hemingway J (1989) A practical field and laboratory manual for the mechanistic detection of insecticide resistance in insects. WHO TDR series book.

[32] Montella IR Martins AJ Viana-Medeiros PF Lima JBP Braga IA Valle D (2007) Insecticide resistance mechanisms of Brazilian *Aedes aegypti* populations from 2001 to 2004: Am J Trop Med Hyg. 77: 467–467. 17827362

[33] Rubert A Guillon-Grammatico L Chandenier J Dimier-Poisson I Desoubeaux G (2016) Insecticide resistance in *Anopheles* mosquitoes: additional obstacles in the battle against malaria. Med Sante Trop. 26: 423–431. 28073732 10.1684/mst.2016.0634

[34] World Health Organization (2012) Global plan for insecticide resistance management in malaria vectors (GPIRM). World Health Organization, Geneva.

[35] Kasap H Kasap M Alptekin D Lüleyap Ü Herath P (2000) Insecticide resistance in *Anopheles sacharovi* Favre in southern Turkey. Bull World Health Org. 78: 687–692. 10859863 PMC2560765

[36] Himeidan YE Chen H Chandre F Donnelly MJ Yan G (2007) Permethrin and DDT resistance in the malaria vector *Anopheles arabiensis* from Eastern Sudan. Am J Trop Med Hyg. 77: 1066– 1068. 18165523

[37] Ganesh K Urmila J Vijayan V (2003) Payrethroid susceptibility and enzyme activity in two malaria vectors, *Anopheles stephensi* (Liston) and *An. culicifacies* (Giles) from Mysore, India. Indian J Med Res. 117: 30–38. 12866824

[38] Vulule J Beach R Atieli F McAllister J Brogdon W Roberts JM Mwangi RW Hawley WA (1999) Elevated oxidase and esterase levels associated with permethrin tolerance in *Anopheles gambiae* from Kenyan villages using permethrin impregnated nets. Med Vet Entomol. 13: 239–244. 10514048 10.1046/j.1365-2915.1999.00177.x

[39] Brogdon WG McAllister JC Corwin AM Cordon-Rosales C (1999). Independent selection of multiple mechanisms for pyrethroid resistance in Guatemalan *Anopheles albimanus* (Diptera: Culicidae). J Med Entomol. 92: 298–302. 10.1093/jee/92.2.298 10333749

[40] Brogdon WG Barber AM (1990) Fenitrothion-deltamethrin cross-resistance conferred by esterases in Guatemalan *Anopheles albimanus*. Pestic Biochem Physiol. 37: 130–139.

[41] Rongnoparut P Boonsuepsakul S Chareonviriyaphap T Thanomsing N (2003) Cloning of cytochrome P450, CYP6P5, and CYP6AA2 from *Anopheles minimus* resistant to deltamethrin. J Vector Ecol. 28: 150–158. 14714662

[42] Hemingway J (2000) The molecular basis of two contrasting metabolic mechanisms of insecticide resistance. Insect Biochem Mol Biol. 30: 1009–1015. 10989287 10.1016/s0965-1748(00)00079-5

[43] Scott J Georghiou G (1986) Malathion-specific resistance in *Anopheles stephensi* from Pakistan. J Am Mosq Control Assoc. 2: 29–32. 3507466

[44] Enayati AA Ladonni (2006) Biochemical assay baseline data of permethrin resistance in *Anopheles stephensi* (Diptera, Culicidae) from Iran. Pakistan J Biol Sci. 9: 1265–1270.

[45] Hemingway J Ranson H (2000) Insecticide resistance in insect vectors of human disease. Annu Rev Entomol. 45: 371–391. 10761582 10.1146/annurev.ento.45.1.371

[46] Limoee M Enayati A Ladonni H Vatandoost H Baseri H Oshaghi (2007) Various mechanisms responsible for permethrin metabolic resistance in seven field-collected strains of the German cockroach from Iran, *Blattella germanica* (L.) (Dictyoptera: Blattellidae). Pestic Biochem Physiol. 87: 138–146.

[47] Hargreaves K Hunt R Brooke B Mthembu J Weeto M Awolola T Coetzee M (2003) *Anopheles arabiensis* and *An. quadriannulatus* resistance to DDT in South Africa. Med Vet Entomol. 17: 417–422. 14651656 10.1111/j.1365-2915.2003.00460.x

[48] Morgan JC Irving H Okedi LM Steven A Wondji CS (2010) Pyrethroid resistance in an *Anopheles funestus* population from Uganda. PLoS One. 5(7): e11872. 20686697 10.1371/journal.pone.0011872 PMC2912372

[49] Corbel V N'guessan R Brengues C Chandre F Djogbenou L Martin T Akogbéto M Hougard JM Rowland M (2007) Multiple insecticide resistance mechanisms in *Anopheles gambiae* and *Culex quinquefasciatus* from Benin, West Africa. Acta Trop. 101: 207–216. 17359927 10.1016/j.actatropica.2007.01.005

